# Reduction of environmental chemicals, toxicity and particulate matter in wet scrubber device to achieve zero emissions

**DOI:** 10.1038/s41598-022-13369-w

**Published:** 2022-06-02

**Authors:** Krishnaraj Ramaswamy, Leta Tesfaye Jule, Nagaprasad N, Kumaran Subramanian, Shanmugam R, Priyanka Dwarampudi L, Venkatesh Seenivasan

**Affiliations:** 1 Mechanical Engineering Department, College of Engineering and Technology, Dambi Dollo University, Dambi Dollo, Ethiopia; 2 Department of Physics, College of Natural and Computational Science, Dambi Dollo University, Dambi Dollo, Ethiopia; 3 Centre for Excellence in Indigenous Knowledge, Innovative Technology Transfer and Entrepreneurship, Dambi Dollo University, Dambi Dollo, Ethiopia; 4 Department of Mechanical Engineering, Ultra College of Engineering and Technology, Madurai, Tamil Nadu 625 107 India; 5 Centre for Drug Discovery and Development, Sathyabama Institue of Science and Technology, Chennai, Tamil Nadu 600119 India; 6 TIFAC, CORE-HD, Department of Pharmacognosy, JSS College of Pharmacy, JSS Academy of Higher Education & Research, Ooty, Nilgiris, Tamil Nadu India; 7 Department of Pharmacognosy, JSS College of Pharmacy, JSS Academy of Higher Education & Research, Ooty, Nilgiris, Tamil Nadu India; 8 Department of Mechanical Engineering, Sri Eshwar College of Engineering, Coimbatore, India

**Keywords:** Environmental chemistry, Environmental impact

## Abstract

The fine particles generated by the foundry industry are present in the atmosphere; they have an impact on the climate because of their influence on atmospheric radioactive phenomena. As a result of this scenario, there is a rising amount of legislation restricting the emission of pollutants from foundry industries and related businesses. In response to this situation, many researchers have concentrated on end-of-pipe technologies, one of which is the wet scrubber, which is a device that is primarily used in foundries to control pollution and is one of the devices that has been incorporated. The disadvantage of using this wet scrubber, on the other hand, is that it contributes to secondary pollution when it is used. In order to combat secondary pollution, a model of an enhanced wet scrubber system that incorporates a multi-sand filtering technology was developed. The performance of this redesigned wet scrubber system was evaluated with the use of computational fluid dynamics (CFD) software. In CFD, the Reynolds stress model was applied for simulation. The pressure magnitudes and velocity magnitudes are obtained by this simulation. The volume fraction of the dust was evaluated through the DPM approach. Because of the introduction of the filtration tank's computation, it was discovered that successful filtration was accomplished using sand filters, meaning that environmental chemicals and particles were totally filtered from 0.17 kg at the entrance to zero kg of particles at the outflow.

## Introduction

The presence of particulate matter (PM) and petite particles is a cause of distress for equally the human health and industrial process^1–5^. PM is a complex mixture consisting of diverse particle types, much of which is likely to cause various adverse effects. For fine particles, there are three types that are used in the industry, i.e., dust capturing mechanisms, namely impaction, interception, and diffusion. By controlling, temperature and humidity could efficiently establish the cleaning process into a single unit. In which toxicity is larger when the lower particle size. Hence the fine particulate matter (PM) can create substantial effects on human health acts both straight and incidentally as a transporter of harmful materials^6–10^. On the other hand, from the emitting sources, the very fine inhalable particles can travel long distances, but once inhaled, they may influence the inmost provinces of the lungs and furthermore can arrive in the circulatory system^11–13^. Also, PM could reduce visibility in cities and also can create large scale effects since it has a stronger influence on atmospheric radioactive phenomena^14–16^. Air pollutants are mainly due to hydrogen chloride (HCl), which is generated from natural chemical reactions and anthropological activities^17^. Moreover, in a natural chemical reaction, NaCl particles can react with HNO_3_ and H_2_SO_4_ to form HCl^18^.

In order to reduce NaCl, the device called wet scrubbers was developed in order to capture very fine particulate matter^19^. The wet scrubbers are mostly utilized in foundries to separate the particulate emission from the furnace. Moreover, the wet scrubbers are utilized in chemical engineering industries, cement industries, textile industries, mining industries, and paper mills to separate the particulate emission from the air/gas stream. In addition, the wet scrubbers are utilized in the circulating fluidized bed (CFB) combustion process to separate the various particulate matters and pollutant gases^20,21^. Later on, we developed a compact wet scrubber working in a self-priming model that consists of several Venturi scrubbers. This compact wet Chandrasekara presented a model that estimates the memory effect of Pollution control using wet scrubbers. Bhave et al.^3^ introduced a wet scrubber suited for small-scale applications which have a wet packed bed scrubber-based producer gas cooling system and cleaning system. Chandrasekara Pillai et al.^6^ researched the possible settings for NO_x_ and SO_2_ subtraction using a scrubber column that was packed with NO–SO_2_–air flue-gas mixtures. The results found that gaseous components were absorbed into the HNO_3_ electrolyte, and Ag (II) mediator acted as an oxidizing agent. It was also observed that removing the ready increase in the presence of SO_2_ and simultaneously gas/liquid and superficial liquid and gas velocity were compared to assess the flow rate effect. Daz-Somoano et al.^8^ evaluated mercury removal efficiency by the influence of different scrubber parameters using thermos-dynamical equilibrium data and laboratory test data. By modifying operational parameters such as pH and slurry can achieve the best results for converting flue gas desulphurization unit to a multi-pollutant control technology which also includes mercury level reduction. Lothgren and Van Bavel^26^ measured the dioxin levels after wet scrubbing systems which had PCDD/Fs on the plastic material and found the levels of dioxin decreased considerably. Perevezentsev et al.^31^ confirmed the extraordinary efficacy of the scrubber column for the depreciation of air polluted with tritiated water vapour and developed a simulation program that reasonably pronounces the process of columns. Biard et al.^4^ investigated the removal of Dimethyl disulphide off columns in an original process compact scrubber that combined an advanced oxidation process. Dimethyl disulphide gas–liquid equilibrium was achieved at the end of the process leading to pollutant reduction. The flue gas cleaning system in the municipal waste incinerator temperature is subject to the absorption/desorption of the materials applied to the wet scrubbing systems. This gas can also be produced during industrial processes such as waste pyrolysis and incineration^20–22^. Even though a major fraction of pollutants such as aerosols are generated by human activities originating from various industrial processes and combustion units, which pose major exposure threats for human beings. Moreover, the risk increases considerably for the habitants of urban areas due to domestic heating, diesel engines emissions and growth of industrial zones near the residential and commercial zones^23–25^. Annular two-phase flow can have complications due to venture geometry which also can express as the gas stream and water in a Venturi. The tube carries the liquid, which journeys as a film laterally, to the wall and the residual amount as gas droplets through the centre of the apparatus, which leads to a continuous exchange between the film and the droplets.

Over the years, numerous models have been proposed to achieve better results by knowing more about hydrodynamics in the scrubber, which may consist of simple correlation analysis or more complex studies such as Computational Fluid Dynamics (CFD) models^26–28^. During the study, it was presumed that pressure drop across the scrubber is due to the alteration in the dewdrops momentum in the entry point of the scrubber and no pressure spent due to the speeding up of the gas and friction between the core and wall of the gas. Designing a more dynamic scrubber, for instance, flue-gas desulfurization (FGD) wet scrubber, needs an in-depth study and consideration of numerous essential factors, which may comprise the scrubber geometry, and gas flow velocity of the tower, pressure changes, SO_2_ exclusion rate, and reagent slurry residue. The main drawback of a wet scrubber is the use of large power for operational needs which is determined by the change in pressure. It is evident from the innovative diagnostic methods like investigation of element size and concentration in gas streams that the particulate matter produced by combustion resources is categorized as per the size of the particle, which may range from a few nanometers up to several microns^29–32^. From the existing emission treatment method^33^, the disposal of the sludge and waste that comes out from the wet scrubber is more complicated. The water under the wet scrubber unit is recirculated to the scrubber continuously. So that the water gets polluted. There is a secondary pollutant formed in the water, which leads to the reduced performance of the wet scrubber. Different factors like throat diameter, its length and the pressure change in atomizers due to different cone angles were considered. Venturi coupled to a holding container, which also acts as a stage extractor, creates the whole structure for the wet scrubber. Later, Gamisans et al.^12^ deliberate the dissemination of the liquid, geometrical effects, flow rates and the self-entrainment by a liquid jet in the ejector-venturi and suggested that similar studies should consider mass transfer theories to fully understand the liquid distribution.

Although many researchers have concentrated on addressing air pollution problems, only very few papers addressed foundry air pollution control using dust collectors^34^, and it is evident that there was no sufficient contribution in addressing secondary pollution reduction in a wet scrubber. An attempt is made to reduce secondary pollution using sand filtering techniques. In practice, the disposal of water and sludge was carried out by a solar evaporation tank. The water gets vaporized by natural sunlight. After vaporization, the sludge deposited on the bottom of the solar evaporation tank was collected and disposed of safely. Since the evaporation process is possible only during daytime and hot summer periods, it is not possible to vaporize the water and collect the sludge during rainy seasons and nighttime. These problems were studied and would like to be implemented in our proposed method by replacing the solar evaporation tank with a sand filter, and a polluted water processing tank was introduced.

“Methodology” validates the model of the proposed wet scrubber by introducing the concept of multi filtration zones along with porosity and zone height with different boundary conditions. “Results and discussion” inspects the results and discusses the effectiveness of wet scrubber with the proposed sand filtration tank using CFD modelling and efficiency by comparing the volume fraction. The previous section reviews all vital themes of the present investigation, its gaps, and eventual research intentions.

## Methodology

**Figure 1:**
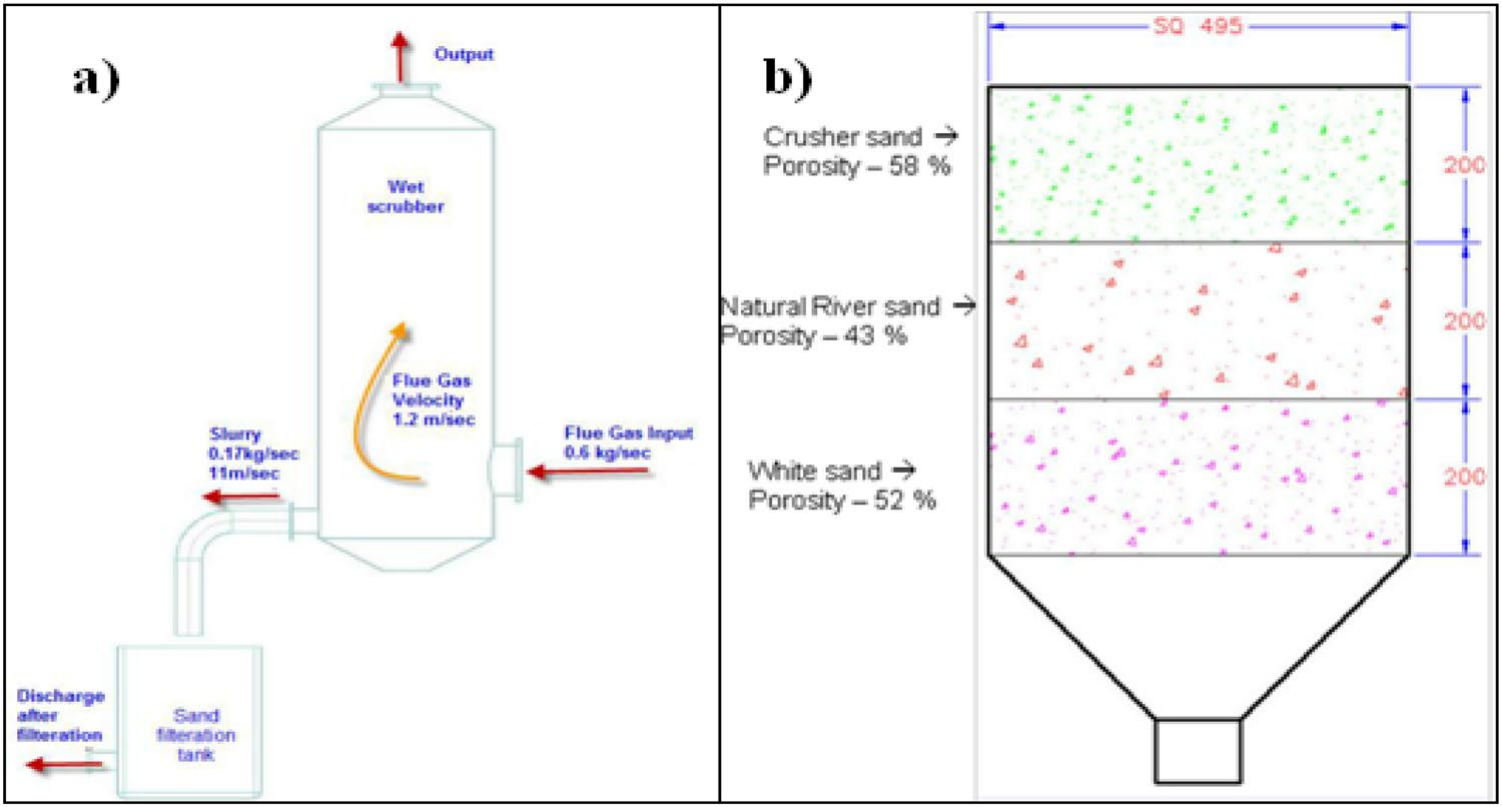
(**a**) Wet scrubber and sand flirtation setup, (**b**) multi filtration zones (porosity and zone height).

A model of wet scrubber, flue gas passage and water pipe connections was created using the CATIA 3D software package. A test was conducted to know the effective particle filtration percentage in the wet scrubber. Step by step procedure was followed to implement the test method. A filtration tank was attached to the wet scrubber in the proposed model to make necessary improvements in the design of the wet scrubber, as shown in Fig. 1a. Skilful and compact test equipment or analytical and simulation software called computational fluid dynamics fluidity, using the commercially available ANSYS software would be imparting to know the dynamic motion of the flow and particles in scrubber its effective flow visible results could be seen theoretically test could manage on the existing model of wet scrubber^35–37^ as well as on the proposed design with boundary conditions. Introducing polluted water processing filtration tank by selecting three types of sand filters for more effective filtering technology, in the form of fine particles, the flue gases were filtered in the wet scrubber.

The input of mass entering into the scrubber unit method is 0.6 kg/s. Generally, the wet scrubber is a pollution control device that reduces the power of the harmful gases coming out from the furnace^38–40^. The treated gas is passed into the chimney, and it is exhausted into the atmosphere. During the wet scrubber process, the air is drained through a mist of water which is created by the nozzle spray; by using separators, water droplets with dust and particles can be removed. Industrial scrubbers are mainly used for removing the potentially harmful pollutants and polluting gas emitted from the various industrial processes. Gases which have the most potent side effects and removal is essential, which include sulphur dioxide (SO_2_) gases from combustion by utilities and industries and a wide variety of by-products and waste gases such as chlorine (Cl_2_), hydrogen chloride (HCl) and hydrogen sulphide (H_2_S)^41^. Initially, in the existing method, the flue gases coming out from the furnace are passed through a duct line. The blower sucks the gases and delivers them into the wet scrubber unit.

The harmful gases coming out of the furnace are most dangerous to exhaust into the atmosphere. Hence, it needs to be treated for exhaust in the atmosphere. There is a barrel of water placed under the wet scrubber unit. The water is pumped into the wet scrubber in two stages. The water was sprayed into the wet scrubber unit. The flue gases were washed by the water on the scrubber unit. The water collects the heavy particles, ashes, and the same barrel.

The same water is recalculated continuously to the wet scrubber unit. The water gets polluted. Secondary pollution was formed in the water. The secondary pollution affects the proposed method. The solar evaporation tank is replaced by a sand filter. Additionally, a polluted water filtration tank was introduced^42–45^. This unit is placed under the wet scrubber unit. Here the polluted water was purified. The sand filters are placed inside the barrel at three different heights. First of all, the sands used in this method are washed thoroughly.

The dirty particle in the sand is removed by this washing. The sand gets purified. Crusher sand is filled in the sand filter at the first stage. Natural river sand is filled in the sand filter at the second stage. The white sand is filled in the sand filters at the third stage, as shown in Fig. 1b. The heavy particles of water and dust that comes out from the wet scrubber unit are passed into the new barrel tank shown in Fig. 1a.

The sludge was stabled on the sand particles in the first stage. The minute particles escaping from the first stage are captured in the second and third stages. So, the water collected at the bottom of the tank was purified. Thus, the secondary pollution was eliminated from the water, and the pure water passed into the wet scrubber unit. The resulting performance of the wet scrubber unit gets improved.

### Boundary condition

**Figure 2:**
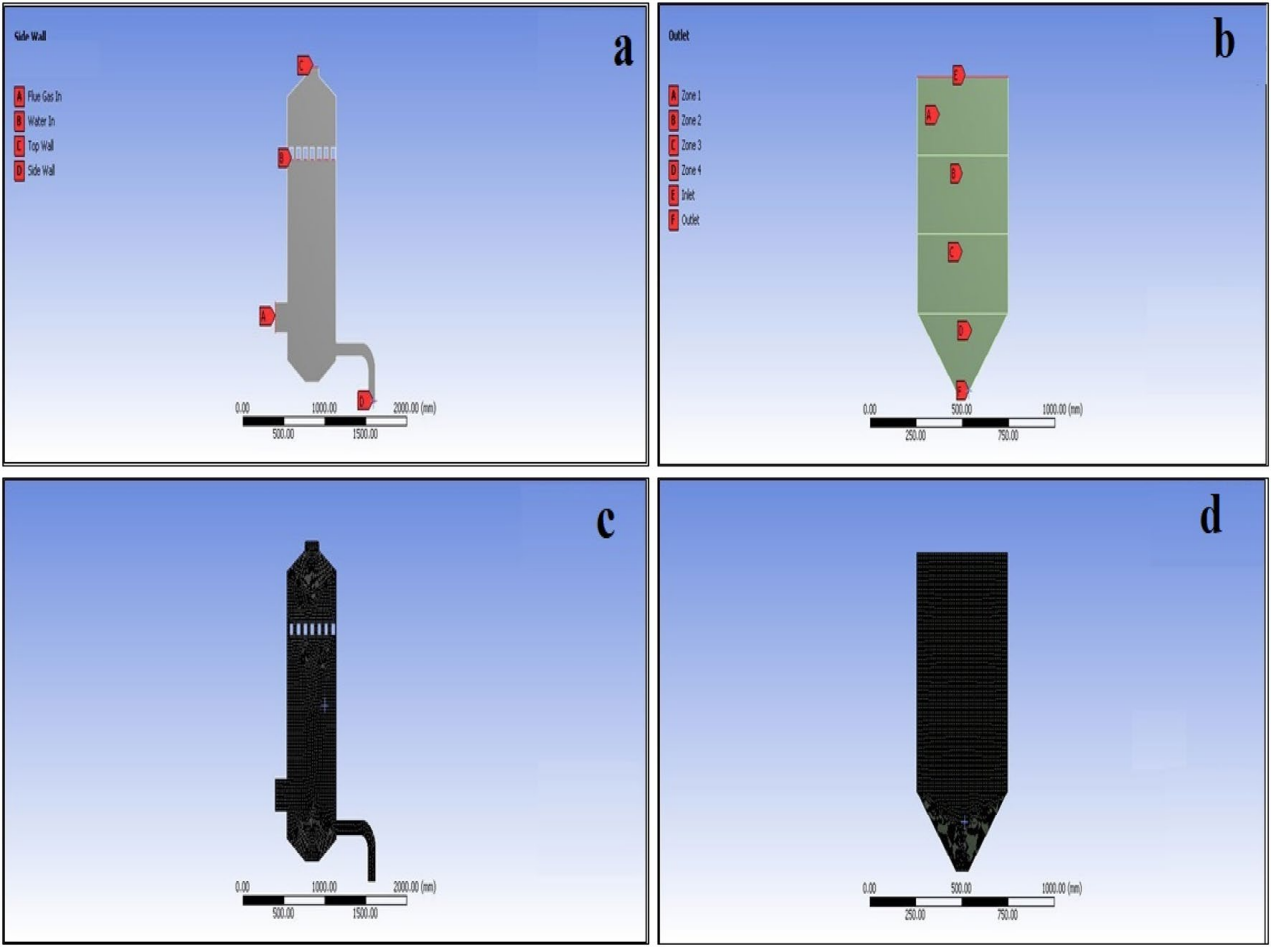
(**a**) Wet scrubber model with secondary water pollution connections, (**b**) filtration tank with sand filtrations zone, (**c**) meshed wet scrubber model, (**d**) meshed filtration tank model.

Table 1 shows the flow medium of the wet scrubber boundary condition in the inlet and outlet. Models Used for wet scrubber CFD analysis has Multiphase (mixture) of flue gas, dust (granular) and water, as shown in Fig. 2a. Table 1 shows the slurry flow parameters and sand porosity used for filtration at different height zones.

**Table 1:** Boundary condition for wet scrubber and multi sand filter.

	Boundary condition	Type	Value
Wet scrubber	Flue gas in	Velocity inlet	1.2 m/s
	Water in	Velocity inlet	5 m/s
	Top wall	Pressure outlet	Ambient (1.01325 bar)
	Side wall	Pressure outlet	Ambient (1.01325 bar)
Multi sand filter	Inlet	Velocity inlet	11 m/s
	Outlet	Pressure Outlet	Ambient (1.01325 bar)
	Zone 1	Porous	Porosity—0.52
	Zone 2	Porous	Porosity—0.48
	Zone 3	Porous	Porosity—0.42
	Injection	Discrete phase	0.17 kg/s

Models Used for sand filtration CFD analysis has a Discrete Phase for Dust Particles, as shown in Fig. 2b. a computation was done with flow parameters, as shown in Table 1.

### Governing equations and computational technique

In this simulation, the Reynolds Stress Model (RSM) was applied to solve the complex flow behaviour. The transient state condition was applied for simulation. The discretization scheme such as SIMPLEC method was established in pressure–velocity pairing. The second-order upwind method was executed for kinetic energy, dissipation rate and momentum for obtaining the exactness in convergence. The time step size was given as 0.001 s. The total number of time steps was given as 1200. The total flow time of this simulation is 1.2 s. The maximum number of iterations per time step was set as 20. In addition, the convergence criterion such as 10^–3^ and 10^–4^ values are set for normalized residuals and scaled residuals for obtaining the accuracy in convergence^47^.

The continuity equation can be written as^47,48^

1 $$\frac{\partial \overline{{u }_{i}}}{\partial {x}_{i}}=0$$

where$\overline{{u }_{i}} \mathrm{is the}$velocity means, ${x}_{i}$ is the position.

The momentum equation can be written as^47,48^

2 $$\frac{\partial \overline{{u }_{i}}}{\partial t}+ \overline{{u }_{j}}\frac{\partial \overline{{u }_{i}}}{\partial {x}_{j}}= -\frac{1}{\rho } \frac{\partial \overline{P}}{\partial {x }_{i}}+v \frac{{\partial }^{2}\overline{{u }_{i}}}{\partial {x}_{j}\partial {x}_{j}}-\frac{\partial }{\partial {x}_{j}}{R}_{ij}$$

where $\rho$ is the gas density, $v$ is the kinematic viscosity of the gas, $\overline{P }$ is the mean pressure, and Reynolds stress tensor can be written as

3 $${R}_{ij}=\overline{{u }_{i}^{^{\prime}}{u}_{j}^{^{\prime}}}$$

where ${u}_{i}^{^{\prime}}$ is the fluctuating velocity component. It can be written as

4 $${u}_{i}^{^{\prime}}= {u}_{i}-\overline{{u }_{i}}$$

The RSTM equation can be written as^47,48^

5 $$\frac{\partial }{\partial t}{R}_{ij}+\overline{{u }_{k}} \frac{\partial }{\partial {x}_{k}}{R}_{ij}= \frac{\partial }{\partial {x}_{k}}\left(\frac{{v}_{t}}{{\sigma }^{k}}\frac{\partial }{\partial {x}_{k}}{R}_{ij}\right)- \left[{R}_{ik }\frac{\partial \overline{{u }_{j}}}{\partial {x}_{k}}+{R}_{ik}\frac{\partial \overline{{u }_{i}}}{\partial {x}_{k}}\right]- {C}_{1}\frac{\varepsilon }{K}\left[{R}_{ij }-\frac{2}{3}{\delta }_{ij}K\right]-{C}_{2}\left[{P}_{ij}-\frac{2}{3}{\delta }_{ij}P\right]-\frac{2}{3}{\delta }_{ij}\varepsilon$$

Turbulence production terms can be written as^47,48^

6 $${P}_{ij}=-\left[{R}_{ik}\frac{\partial \overline{{u }_{j}}}{\partial {x}_{k}}+{R}_{jk}\frac{\partial \overline{{u }_{i}}}{\partial {x}_{k}} \right], P=\frac{1}{2} {P}_{ij}$$

where ${v}_{t}$ is the turbulent viscosity, P is the kinetic energy production (fluctuation).

Turbulence dissipation rate, ε can be written as^47,48^:

7 $$\frac{\partial \varepsilon }{\partial t}+ \overline{{u }_{j}}\frac{\partial \varepsilon }{\partial {x}_{j}}=\frac{\partial }{\partial {x}_{j}}\left[\left(v +\frac{{v}_{t}}{{\sigma }^{\varepsilon }}\right) \frac{\partial \varepsilon }{\partial {x}_{j}}\right]-{C}^{\varepsilon 1}\frac{\varepsilon }{K}{R}_{ij}\frac{\partial \overline{{u }_{i}}}{\partial {x}_{j}}-{C}^{\varepsilon 2}\frac{{\varepsilon }^{2}}{K}$$

The fluctuating kinetic energy can be written as:

8 $$K=\frac{1}{2} \overline{{u }_{i}^{^{\prime}}{u}_{i}^{^{\prime}}}$$

The constants are ${\sigma }^{k}=1$, ${\sigma }^{\varepsilon }=1.3$, C^ε1^ = 1.44, C_1_ = 1.8, C_2_ = 0.6, and C^ε2^ = 1.92.

The particle motion equation can be written as^47,48^

9 $$\frac{{du}_{Pi}}{dt}={F}_{D}\left({u}_{i}-{u}_{Pi}\right)+\frac{{g}_{x}({\rho }_{P}-\rho )}{{\rho }_{P}}$$

10 $$\frac{{dx}_{Pi}}{dt}={u}_{Pi}$$

11 $${F}_{D}=\frac{18\mu }{{\rho }_{P}{d}_{P}^{2}}\frac{{C}_{D}{R}_{e}}{24}$$

12 $${R}_{e}=\frac{{\rho d}_{P }\left|{u}_{P}-u\right|}{\mu }$$

### Model validation

The dust particles present in the flue gas were toxic, and it also has carcinogenic components^46^. Hence secondary filtration is carried out by spraying the water with a velocity of 5 m/s through the water processing system. Flue gas velocity is considered as 1.2 m/s. Springing action trapped the fine particles and fall down due to gravity and collected in the filtration tank through the outlet pipe set up in the wet scrubber, for which a processing tank for better trapping is introduced in the wet scrubber system, and the input is the same as in the existing method and output has been set up after at the processing tank, collected waste without a slurry. The fine particles enter into the wet scrubber, particles are partially trapped inside the wall, and the remaining filtering is done at the filtration tank while particles are passing through it. An automatic unstructured mesh was generated using the ANSYS workbench for the wet scrubber and sand filter mixed element type, as listed in Table 2.

**Table 2:** Building a mesh for the wet scrubber and sand filter model.

Model	Element type	No of Elements
Wet scrubber	Mixed (hex & tri)	46,150
Sand filter	Mixed (hex & tri)	77,260

Meshing is the process of splitting the computational domain. Figure 2c shows the meshed model of the wet scrubber; the wet scrubber model was split into 46,150 elements with mixed element types, i.e. with both Hexa and tri elements. Figure 2d shows the meshed model of the filtration tank; the filtration tank model was split into 77,260 elements with mixed element type, i.e. with both Hexa and tri elements.

The gird independence study (GIS) was executed to validate the mesh quality. In GIS, five types of mesh, such as 44,150, 44,650, 45,150, 45,650 and 46,150, were generated for the wet scrubber to validate the mesh quality. For the sand filter, five types of the mesh, such as 75,260, 75,760, 76,260, 76,760 and 77,260, were generated to validate the quality of the grid. One of the output parameters, such as maximum pressure, was considered for validating the mesh size. It was found that the maximum error percentage obtained between each mesh size is less than 1%. It indicates that the generated mesh is in excellent condition. However, for avoiding the computational uncertainty largest mesh size was selected for simulation.

## Results and discussion

The primary objective of the present study is to inspect the effectiveness of wet scrubber with the proposed sand filtration tank using CFD modelling and efficiency by comparing the volume fraction.

### The volume fraction of dust

**Figure 3:**
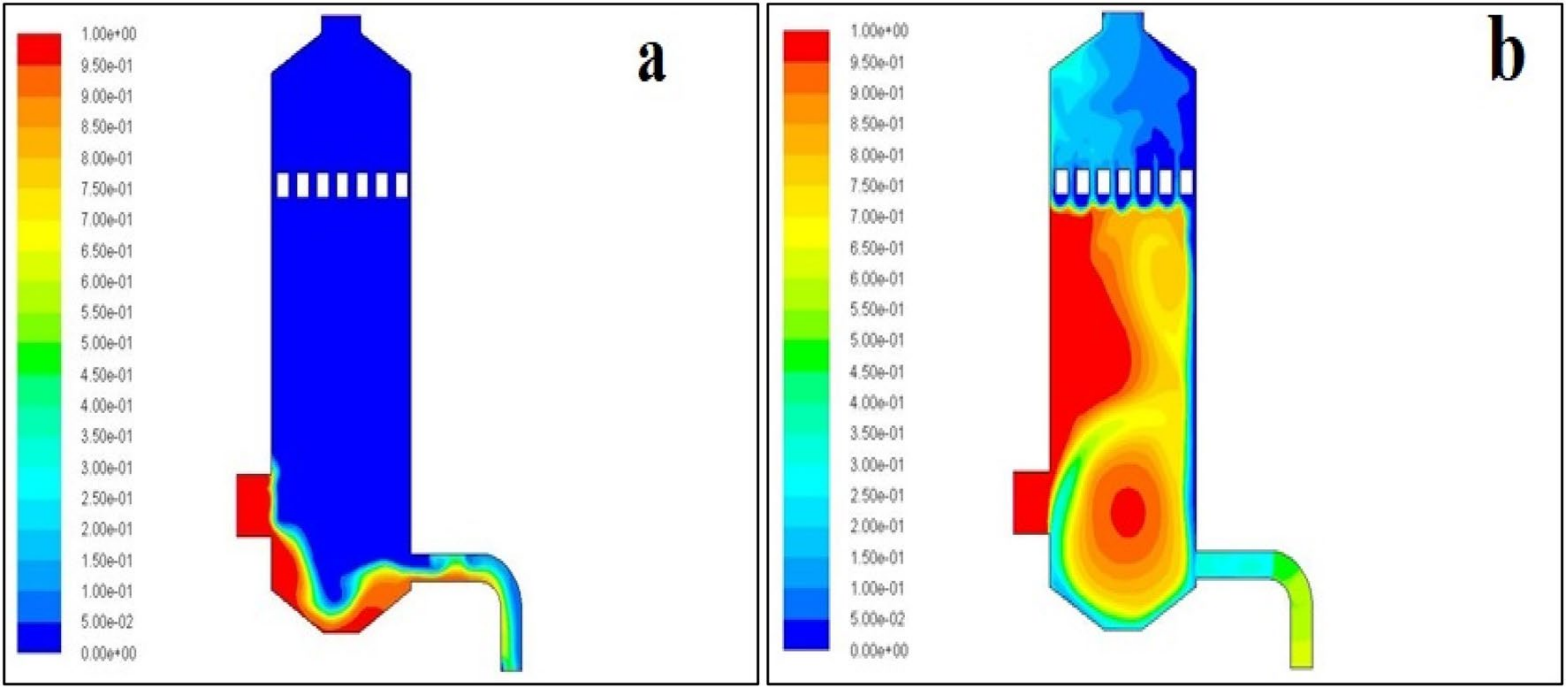
(**a**) Volume fraction of dust, (**b**) volume fraction of flue gas.

In the simulation, dust particles were considered to be in the granular phase. In ANSYS FLUENT, we can set the collision between two phases (water and dust particles). Below Fig. 3a. Shows the volume fraction (percentage) of the granular phase (dust particles). Due to the interaction of water and dust particles, the dust particles settled at the bottom of the scrubber. Usually, less dense particles move in the upward direction, and high dense particles move in the downward direction. In this simulation, the water droplets sprayed against the moving dust particles stream. Therefore, the density of the dust particles was increased. Therefore, the sprayed water droplets pick up the flying dust particles, and finally, the particulate matters are settled at the bottom of the wet scrubber. Some of the dust particles went out along with water through the side outlet. The blue and red colour indicates the minimum (zero) and maximum (100%) volume fraction of the dust particles in the scrubber. The maximum volume fraction is at the bottom of the scrubber, which indicates the deposition of dust particles due to the interaction (collision) with the water^35–38^.

### Surface integral of dust volume fraction

**Figure 4:**
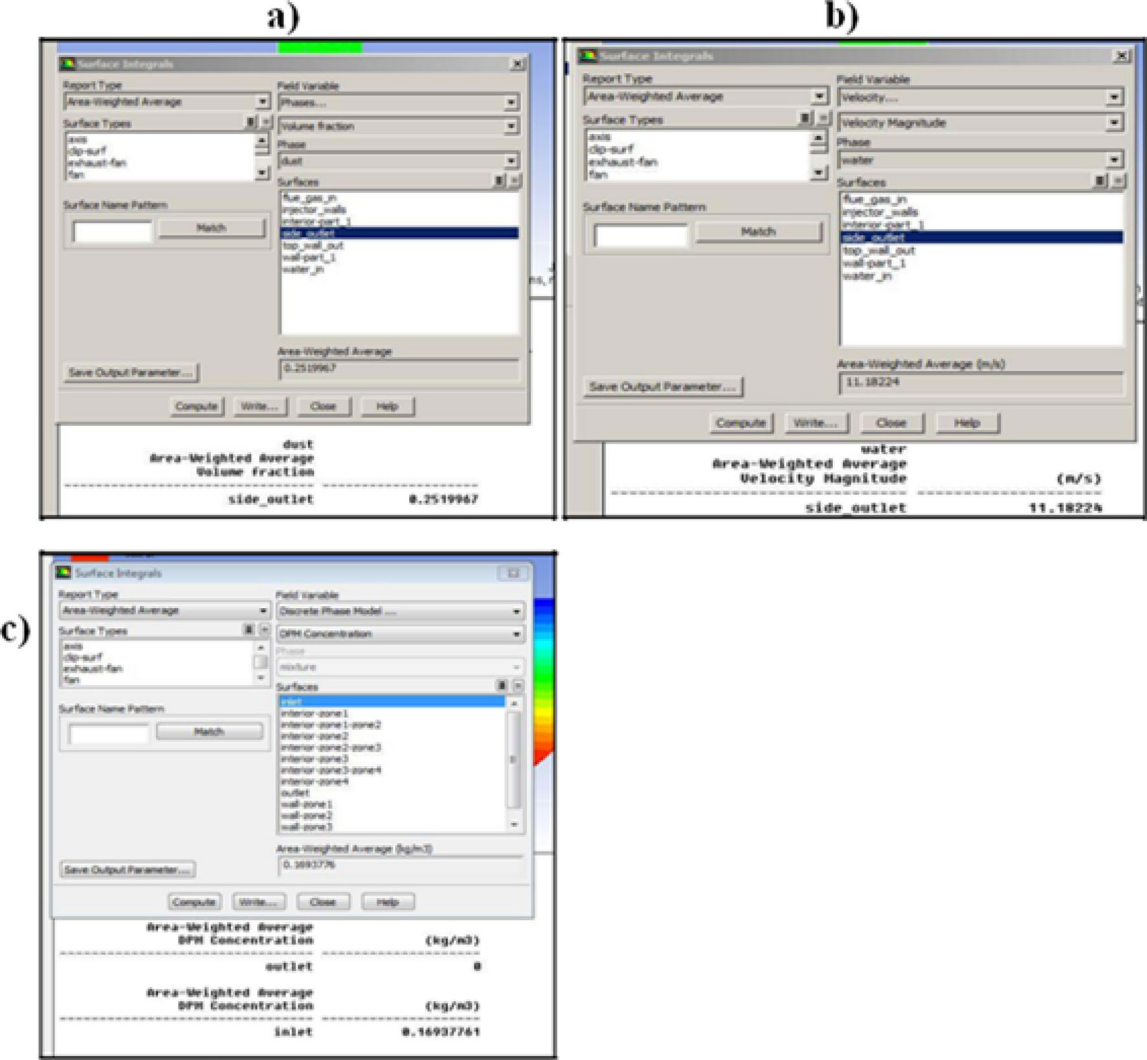
(**a**) Area weighted average of the volume of (**a**) fraction of dust, (**b**) flue gas, (**c**) DPM concentration.

Area weighted average as shown in Fig. 4a. Presents the outcome of the area-weighted average computation over all the selected surfaces. An area-weighted average result for the volume fraction of dust particles was taken to check the amount of dust particles present in the inlet and outlet of the wet scrubber. Approximately 25 percent of inlet dust particles come out. i.e. around 0.17 kg/s (inlet flow rate = 0.6 kg/s).

### The volume fraction of flue gas

The concentration of flue gas in the wet scrubber is shown in Fig. 3b. It clearly shows that flue gas escapes through the water injection port. The red colour shows the maximum (100%) concentration, and the blue colour indicates the minimum (zero) flue gas concentration.

### The velocity of water at the outlet

The surface integral of the area-weighted average velocity magnitude of the water at the side outlet is shown in Fig. 4b. This outlet velocity will be used as the inlet velocity for the sand filter. The water from the wet scrubber comes out with an average velocity of 11.18 m/s.

### Output parameter of wet scrubber

Quantity of Dust particle at the outlet – 0.17 kg/s (25% of the inlet valve). The velocity of water at the outlet is 11 m/s. The above parameter will be used as an inlet parameter for sand filtration.

### Pressure contours in sand filtration

**Figure 5:**
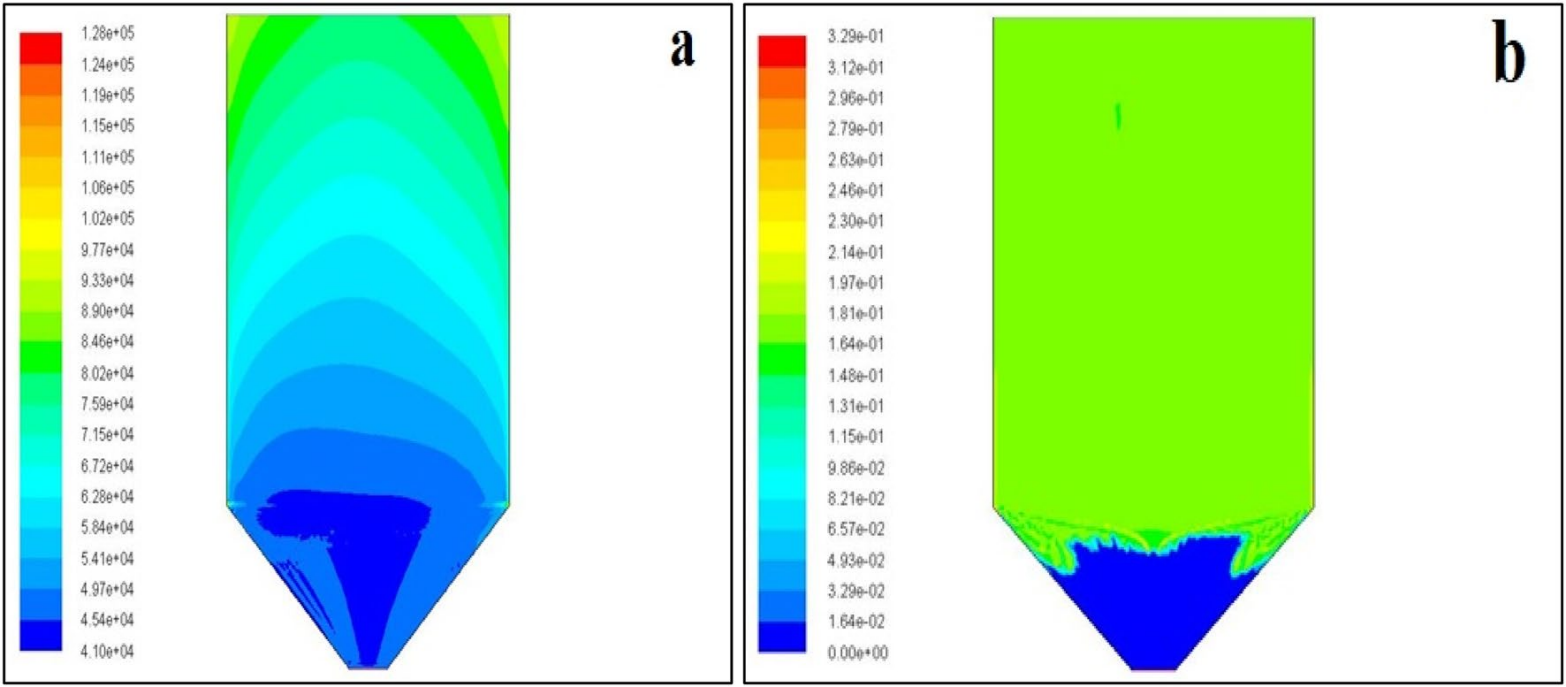
(**a**) Total pressure contours, (**b**) discrete phase concentration.

The liquid in the filter can be pushed through by creating a difference in pressure between the inlet and outlet sides of the filter. This pressure differential is greatly subjective to the resistance of the flow of the filter or medium, from Fig. 5a. Blue and red colours in the contours indicate the minimum (41,000 Pascal) and maximum (128,000 Pascal) value of pressure, respectively. From the colour contours, it is visually predicted that there is a high-pressure drop (87,000 Pascal) between the inlet and outlet, which means an effective filtration has happened. These contour plots indicate that the pressure drop was decreased at the bottom of the wet scrubber. Furthermore, it increases towards the top roof of the wet scrubber. The velocity of the moving particles is completely reduced due to this less pressure drop. Therefore, the dust particles are settled at the bottom due to this less pressure drop at the conical section of the wet scrubber. Moreover, spraying the water particles increases the density of the particles it decreases the velocity of the dust particles. Thus, it restricts the particles moving toward the atmosphere.

### Discrete phase concentration

In the simulation, dust particles of size 1 micron were considered as a discrete phase. ANSYS FLUENT model described the magnitude of the interphase exchange of momentum, heat, and mass in the individual control volume. It is also able to analyze the total concentration of particles present in the designated discrete phase. Below, Fig. 5b shows the concentration of the discrete phase (dust particles). Due to the resistance of the porous medium (sand filter), the particle concentration was high (0.16 kg) in the sand filters area, and it was zero at the outlet. The green colour in the figure indicates the concentration of the discrete phase (dust particles). Since the variation of the discrete phase is less throughout the height of the sand filter, the concentration contours are visualized as uniform.

### Surface integral of Discrete Phase Model (DPM) concentration

Figure 4c displays the result of the area-weighted average computation over all the selected surfaces. An area-weighted average result for discrete phase concentration was taken to check the amount of dust particles present in the inlet and outlet of the sand filter. The below surface integral result shows that effective filtration was done, i.e. dust particle was completely filtered from 0.17 kg at the inlet to zero at the outlet.

## Conclusion

The wet scrubber unit's performance mainly depends on the purity of water. Therefore, a good Improvement in the wet scrubber is mandatory. To achieve better performance of a wet scrubber, new conditions to eliminate the secondary pollution formed on the wet scrubber is proposed. To eliminate the secondary pollution, a filtration tank was fitted at the outlet of the wet scrubber. The multiphase model was used to simulate the deposition and escaping of dust particles in the wet scrubber unit. Then the simulation of the filtration tank was done using the discrete phase model. From the simulation, the volume fraction of the dust particle present in the wet scrubber and at the outlet of the unit was measured. The outlet of the wet scrubber unit contains 0.17 kg of dust particles; it is about 25% (0.6 kg) of inlet dust particle concentration. Also, the outlet velocity of the water in the wet scrubber unit was measured as 11.18 m/s. Then the computation of the filtration tank was done using the wet scrubber outlet parameter. The filtration tank was simulated as a porous medium. Effective filtration was identified by measuring the dust particle concentration at the inlet and outlet of the filtration tank. From the computation of the filtration tank, it was found that effective filtration was done using sand filters, i.e. environmental chemicals and particle matter were completely filtered from 0.17 kg at the inlet to zero at the outlet.

## Data Availability

The data are included with in the article.
